# Individual, facility and policy level influences on national coverage estimates for intermittent preventive treatment of malaria in pregnancy in Tanzania

**DOI:** 10.1186/1475-2875-7-260

**Published:** 2008-12-18

**Authors:** Tanya Marchant, Rose Nathan, Caroline Jones, Hadji Mponda, Jane Bruce, Yovitha Sedekia, Joanna Schellenberg, Hassan Mshinda, Kara Hanson

**Affiliations:** 1 Department of Public Health and Policy, London School of Hygiene and Tropical Medicine, Keppel Street, London, UK; 2 Department of Infectious and Tropical Diseases, London School of Hygiene and Tropical Medicine, Keppel Street, London, UK; 3 Ifakara Health Institute, PO Box 78373, Mikocheni, Dar es Salaam, Tanzania

## Abstract

**Background:**

Delivery of two doses of intermittent preventive treatment of malaria during pregnancy (IPTp) is a key strategy to reduce the burden of malaria in pregnancy in sub-Saharan Africa. However, different settings have reported coverage levels well below the target 80%. Antenatal implementation guidelines in Tanzania recommend IPTp first dose to be given at the second antenatal visit, and second dose at the third visit. This investigation measured coverage of IPTp at national level in Tanzania and examined the role of individual, facility, and policy level influences on achieved coverage.

**Methods:**

Three national household and linked reproductive and child health (RCH) facility surveys were conducted July-August 2005, 2006, and 2007 in 210 clusters sampled using two-stage cluster sampling from 21 randomly selected districts. Female residents who reported a livebirth in the previous year were asked questions about malaria prevention during that pregnancy and individual characteristics including education, pregnancy history, and marital status. The RCH facility serving each cluster was also surveyed, and information collected about drug stocks, health education delivery, and the timing of antenatal care delivery by clinic users.

**Results:**

The national IPTp coverage had declined over the survey period being 71% for first dose in 2005 falling to 65% in 2007 (χ^2 ^2.9, p = 0.05), and 38% for second dose in 2005 but 30% in 2007 (χ^2 ^4.4, p = 0.01). There was no evidence of any individual factors being associated with second dose coverage beyond living in an urban area. Availability of sulphadoxine-pyrimethamine at RCH had decreased year on year from 85% of clinics in stock in 2005 to 60% in 2007 (χ^2 ^20.6, p < 0.001). This reduction was evident in rural but not urban clinics. If safety recommendations and national antenatal care guidelines for IPTp delivery were followed, in 2007 only 76% of pregnant women could have received IPTp first dose and only 46% could have received second dose.

**Conclusion:**

There is scope to improve IPTp first and second dose coverage at national scale within existing systems by improving stock at RCH, and by revising the existing guidelines to recommend delivery of IPTp after quickening, rather than at a pre-defined antenatal visit.

## Background

Over the last decade a number of reviews have firmly established the burden of malaria in pregnancy as a priority public health issue [1-4]. One intervention widely promoted to tackle this burden in malaria endemic areas in sub-Saharan Africa is intermittent preventive treatment for pregnant women (IPTp) [5,6].

With few exceptions, the implementation of IPTp has been integrated within the existing antenatal care structure as part of the WHO recommended focused antenatal care schedule [7]. The regime consists of the administration of two or three complete curative doses of an anti-malarial medicine, the first after quickening – early in the second trimester – and subsequent doses at monthly intervals [8]. There is increasing awareness of the importance of administering at least two doses for a protective effect [9-12]. Currently, sulphadoxine-pyrimethamine (SP) is the recommended IPTp drug in several African countries although, in light of increasing malaria parasite resistance to SP, alternative drugs are under investigation [13,14].

It is estimated that 80% of women across sub-Saharan Africa access antenatal clinic at least once during pregnancy, and 74% at least twice [15]. SP is a relatively cheap drug, has been widely available, and is amenable to directly observed therapy requiring only a single dose for each treatment. In theory, therefore, IPTp coverage using SP should be high. However, as reported from many settings [16-21] second dose coverage has been disappointing, with one exception [22], and there is concern that the effectiveness of IPTp under programme conditions may be much reduced [23]. There is an urgent need to understand how the potential of this strategy can be realized.

Using national level linked household and facility data in Tanzania collected annually over the period 2005–2007, trends in IPTp coverage at a national scale are investigated, and the influence of individual, facility and policy level indicators on second dose IPTp coverage countrywide explored.

## Methods

### Study setting

The mainland of the United Republic of Tanzania, which excludes Zanzibar, has 133 districts across 21 regions and a population of over 33 million people. It is a country with high fertility and good access to antenatal care with over 95% of women having at least two antenatal visits [24]. The country is highly endemic for malaria and IPTp has been implemented as national policy for the last five years. The antenatal implementation schedule offers SP to all pregnant women attending antenatal clinics at their second visit between 20 and 24 weeks gestation for the first dose and at their third visit between 28 and 32 weeks for the second dose [25].

### Study design

This investigation uses data collected as part of the Tanzanian National Voucher Scheme (TNVS) monitoring and evaluation conducted in July-August of 2005, 2006 and 2007, full details of which, including survey instruments used, are available elsewhere [26]. Three data sources are analysed: household surveys, reproductive and child health (RCH) facility surveys, and RCH user surveys.

(1) Household surveys were conducted in the same randomly selected 21 districts across mainland Tanzania in July/August each year with a two-stage cluster sample design. Ten clusters (wards) were selected with probability proportional to population size within each district. For each cluster a sub-village (*kitongoji*) was sampled with simple random sampling. Finally, 30 households were sampled from each cluster using a EPI sampling approach.

At each household, interviews were conducted in Kiswahili with the household head, with all women aged 15–49 and with all caregivers of children under five. An additional interview schedule was applied for women aged 15–49 who were currently pregnant, or who had had a live birth in the preceding year. These data are used to provide estimates on IPTp coverage and individual level influences.

(2) The RCH facility serving each cluster (described above) was selected for survey. At each facility, equipment and supplies were checked, staff and services provided recorded, health education sessions observed, and a review of routine antenatal record keeping was conducted. These data are used to explore the facility level characteristics influencing IPTp coverage estimates.

(3) At each RCH facility, up to a maximum of 7 women who accessed antenatal care on the day of survey were invited to be interviewed. The interview schedule included questions about timing of attendance to RCH throughout pregnancy, the timing of intervention delivery, and knowledge of specific aspects of malaria and reproductive health. These data are used to explore the policy level (specifically timing) characteristics influencing IPTp coverage estimates.

### Data analysis and definitions

IPTp coverage was measured in the household survey. The definition applied was in line with that recommended by Roll Back Malaria [27]: "The proportion of all women surveyed who had a live birth in the last year who reported having received one (*or at least two where stated*) doses of IPTp during their last pregnancy."

The residential status of households is defined at the Ward level from the 2002 Tanzanian National Census, grouped as urban and non-urban (rural/semi-urban).

The socio-economic status of households relative to each other is grouped by quintile, quintile 1 being most poor. This was derived from principal components analysis using a combination of variables including household head education level, housing conditions, asset ownership of household and whether the house was rented or not.

All estimates were adjusted for cluster sampling using svy commands in STATA 10. The χ^2 ^test was used to examine differences in IPTp coverage by explanatory variables within survey years, and to examine differences in IPTp coverage levels across survey years. A summary of respondents from the household and facility surveys included in this analysis is given in Table 1.

**Table 1:** Respondents to Household, RCH facility and RCH facility user surveys, Tanzanian National Voucher Scheme 2005–2007

Year	2005	2006	2007
TNVS Annual Survey Sample			
N. Districts	21	21	21
N. Clusters	210	210	210
Household Survey			
N. Households	6199	6260	6198
N. Women with a live birth in previous 12 months	1171	1229	1214
RCH facility survey			
N. Clinics	190	188	192
N. Clinics providing outreach services	108	100	122
N. RCH facility user interviews	848	862	914

### Ethical approval

The monitoring and evaluation protocol was approved by the ethical review committee at London School of Hygiene and Tropical Medicine, UK, and by the Institutional Review Board at Ifakara Health Institute, Tanzania. The purpose and potential risks arising from participation were explained to all sampled persons, and anonymity was assured. Each interviewee was asked to provide written consent.

## Results

### Individual level characteristics

#### Coverage of IPTp across time

Estimates for both first and second dose coverage of IPTp have declined over the period from 71% (836/1,171) in 2005 to 65% (791/1,214) in 2007 reporting first dose (χ^2 ^2.9, p = 0.05) and 38% (443/1,171) in 2005 to 31% (370/1,214) in 2007 (χ^2 ^4.4, p0.01) reporting second dose (Table 2).

**Table 2:** Percent of women reporting that they received first or second dose of an anti-malarial drug as intermittent preventive treatment (IPTp) at RCH facilities in Tanzania, TNVS Household survey 2005–2007

	First dose % (95% CI)	χ^2^ (p-value)	Second dose % (95% CI)	χ^2^ (p-value)
***For three survey years:***				
TNVS Household survey 2005 [N = 1171]	71.4 (67.8–74.7)		37.8 (34.3–41.5)	
TNVS Household survey 2006 [N = 1229]	68.6 (65.1–71.9)		35.2 (31.7–38.9)	
TNVS Household survey 2007 [N = 1214]	65.2 (61.3–68.9)	2.9 (0.05)	30.5 (27.2–34.2)	4.4 (0.01)
***For 2007 only:***				
**Residence**				
Urban [N = 95]	77.8 (66.1–86.4)		44.2 (31.7–57.4)	
Non-urban [N = 1119]	64.2 (60.0–68.1)	4.8 (0.02)	29.4 (22.9–33.1)	5.1 (0.02)
**Education level**				
No education [N = 298]	54.7 (48.4–60.8)		26.8 (21.2–33.3)	
Incomplete primary [N = 147]	59.2 (51.4–66.5)		29.9 (23.4–37.4)	
Complete primary [N = 728]	69.9 (65.6–73.9)		31.6 (27.6–35.9)	
Secondary + [N = 41]	80.5 (65.1–90.1)	10.2( < 0.01)	41.5 (25.6–59.2)	1.3 (0.2)
**Marital Status**				
Married/co-habit [N = 1005]	64.4 (59.9–68.5)		30.5 (27.0–34.3)	
Previously married [N = 101]	72.3 (62.4–80.4)		34.6 (26.4–43.9)	
Never married [N = 108]	66.7 (56.2–75.7)	1.1 (0.3)	26.8 (18.3–37.6)	0.7 (0.4)
**Age**				
< 20 years [N = 149]	62.4 (53.9–70.2)		29.5 (22.6–37.5)	
20–24 [N = 316]	65.2 (58.9–70.9)		32.9 (27.1–39.3)	
25–29 [N = 279]	68.4 (61.8–74.4)		32.3 (26.3–38.9)	
30–34 [N = 238]	64.3 (57.0–70.9)		30.2 (24.6–36.5)	
35–39 [N = 147]	63.3 (54.6–71.1)		30.5 (20.3–43.2)	
40+ [N = 72]	72.2 (58.9–82.5)	0.6 (0.6)	25.8 (18.9–34.3)	0.5 (0.7)
**Socio-economic status**				
Q1 (Most poor) [N = 208]	55.3 (47.7–62.6)		26.9 (20.9–33.8)	
Q2 [N = 291]	60.5 (53.6–66.9)		26.8 (21.4–32.9)	
Q3 [N = 244]	67.2 (60.6–73.2)		31.1 (24.7–38.3)	
Q4 [N = 255]	67.4 (61.2–73.1)		32.5 (26.8–38.8)	
Q5 (Least poor) [N = 371]	76.7 (69.5–82.6)	5.9 (< 0.01)	36.3 (28.9–44.4)	1.5 (0.18)

#### Coverage of IPTp by individual characteristics

Coverage was disaggregated for 2007 by the individual level characteristics of age, marital status, education level of the woman, household socio-economic status and residence (Table 2). Residence was highlighted as an important differential for IPTp first and second dose estimates, coverage being higher amongst women in urban than women in non-urban settings for first dose: 78% (74/95) compared to 64% (718/1119), χ^2 ^4.8 (p = 0.02) and for second dose: 44% (42/95) urban compared to 29% (329/1119) non-urban, χ^2 ^5.1(p = 0.02). There was evidence to suggest that women with education levels beyond completed primary, and more wealthy women, were more successful than other women in getting a first dose of IPTp but this was not true for second dose (Table 2).

#### Reasons for not taking IPTp

Women who reported attending an antenatal clinic when they were pregnant, who said they had not received the first dose of IPTp, were asked why they had not. Over 90% reported that they had not been asked whether they wanted it, with a slight increase in the percent not asked by survey year: 2005: 92.0% (307/334), 2006: 92.5% (355/384), and 2007: 96.2% (406/422) (χ^2 ^2.8, p0.06). This finding does not appear to be linked to individual status for any of the survey years, with no difference in the percent not asked by socio-economic quintile (χ^2 ^2.0, p0.7), age (χ^2 ^4.0, p0.5), education (χ^2 ^2.2, p0.5) or by residence (χ^2 ^0.3, p0.8) of the woman in 2007.

### Facility level characteristics

#### Stock of sulphadoxine/pyrimethamine at antenatal clinic

There was a decline in the number of RCH facilities with SP in stock on the day of the survey from 85% (161/190) in 2005 to 59% (78/192) in 2007 (χ^2 ^20.6, p < 0.001) across all levels of clinics (Table 3). This aggregate figure was dominated by declines in rural clinics from 84% (105/125) in 2005 to 55% (73/133) in 2007 (χ^2 ^16.5, p < 0.001), but there was no difference in urban clinics, being 86% (19/22) in 2005 and 88% (15/17) in 2007 (χ^2 ^1.1 (p = 0.2).

**Table 3:** Stock of sulphadoxine pyrimethamine on the day of survey, TNVS RCH Facility Survey 2005–07

	2005	2006	2007	χ^2 ^(p-value)^1^
**All**	84.7 (78.8–89.2) [N = 190]	74.5 (67.7–80.2) [N = 188]	59.5 (52.2–66.1) [N = 192]	20.6 (< 0.001)
**By level of facility**				
Dispensary	84.7 (77.9–89.6) [N = 150]	71.3 (63.1–78.3) [N = 136]	55.6 (47.3–63.5) [N = 144]	19.3 (< 0.001)
Health Centre	95.2 (72.5–99.3) [N = 21]	80.6 (62.9–91.1) [N = 31]	72.4 (53.6–85.6) [N = 29]	3.2 (0.04)
Hospital	73.7 (50.0–88.7) [N = 19]	85.7 (63.6–95.3) [N = 21]	68.4 (44.9–85.2) [N = 19]	0.9 (0.3)
χ^2 ^(p-value)^2^	1.7 (0.1)	1.3 (0.2)	1.7 (0.1)	
**By residence**				
Rural	84.0 (76.4–89.5) [N = 125]	72.3 (63.9–79.4) [N = 130]	54.9 (46.3–63.2) [N = 133]	16.5 (< 0.001)
S-Urban	86.0 (72.1–93.6) [N = 43]	70.7 (55.0–82.6) [N = 41]	61.9 (46.4–75.3) [N = 42]	3.5 (0.03)
Urban	86.4 (64.9–95.6) [N = 22]	100 [N = 17]	88.2 (62.9–97.1) [N = 17]	1.2 (0.2)
χ^2 ^(p-value)^2^	0.07 (0.9)	3.3 (0.03)	3.5 (0.03)	

χ^2 ^(p-value)^1 ^for difference between survey years

χ^2 ^(p-value)^2 ^for difference within survey years

This cross-sectional data reports on stock on the day of survey but not duration of stock-out. However, by linking the coverage estimates derived from RCH facility user interviews with the stock in the facility that day we are able to demonstrate the impact of stock-outs on access. In 2007, first dose IPTp coverage was only 41% (49/121) amongst women accessing a facility experiencing a SP stock-out, compared to 89% (164/184) amongst women accessing a facility with SP stock that day (χ^2 ^54.3 (< 0.001).

#### Visual aides for IPTp

Not only was there a significant decline in SP stocks at RCH facilities but there was also a decline in the percent of facilities displaying posters explaining the purpose and benefits of IPTp from 70% (134/190) in 2005 to 50% (96/191) in 2007 (χ^2 ^11.5, p < 0.001).

#### IPTp at outreach clinic

Around 60% of RCH clinics reported providing an outreach antenatal service to women in remote areas (Table 1). A decline was observed in the percent of facilities administering IPTp at outreach clinic from 63% (68/108) in 2005 to 45% 55/122 in 2007 (χ^2 ^6.5, p0.001).

#### Health education

Health education sessions were observed in 54% (111/190) of facilities in 2005, 67% (126/188) in 2006 and 62% (118/191) in 2007. A check list was used to indicate which topics were mentioned during the observed session. Just under half of all sessions delivered malaria prevention messages, with no difference by year: 47% in 2005, 43% in 2006 and 46% in 2007 (χ^2 ^0.2, p0.8).

In 2006, as part of the RCH facility antenatal user interview, an exploration was made into pregnant women's understanding of malaria prevention options. 66% (571/862) were unable to answer the question "How many times should you take the medicine to prevent malaria during pregnancy?" and a further 6% (49/862) answered only one dose.

### Policy level

#### IPT schedule for delivery

Pregnant women in Tanzania are recommended to first attend antenatal clinic by 16 weeks gestation, their second visit to take place around 20 weeks, and third around 28 weeks. In this population however (and typical of sub-Saharan Africa) the median gestation at first visit was 20 weeks and the median gestation at second visit was 26 weeks. In reality, therefore, women attend clinic later than recommended antenatal policy and this is found to disrupt the antenatal schedule for delivery of IPTp which recommends delivery of IPTp first dose at the second antenatal visit.

**Figure 1:**
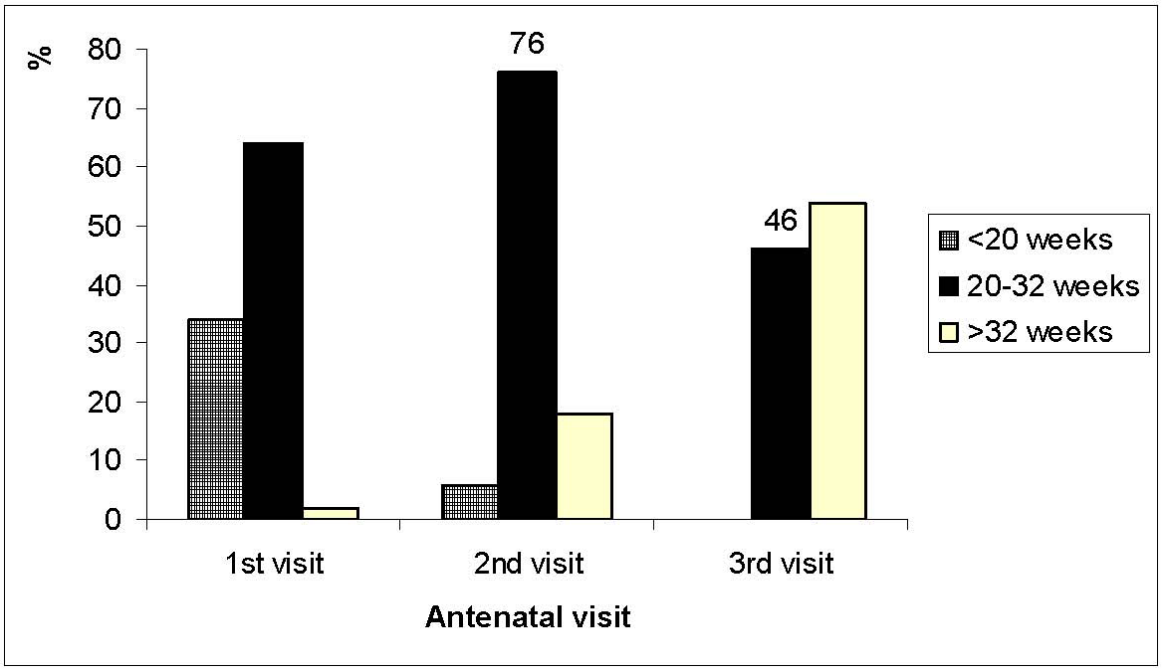
Distribution of antenatal attendance < 20 weeks (too early IPTp), 20–32 weeks (recommended IPTp) and > 32 weeks (too late IPTp) gestation at first, second and third antenatal visits to RCH facility, TNVS RCH facility survey, 2007.

In Figure 1, the gestation of women at first, second, and third antenatal visit is grouped according to implication for IPTp delivery (< 20 weeks: too early; 20–32 weeks: recommended; > 32 weeks: too late). At the first visit, 64% of pregnant women were within the recommended gestation to receive IPTp yet current policy recommends IPTp be given at second visit, and potentially these women were missed. At the second visit, 76% of pregnant women were within the recommended gestation to receive IPTp in accordance with policy. At the third visit, only 46% of pregnant women were within the recommended gestation to receive IPTp second dose in accordance with antenatal policy. These figures are not dissimilar to the coverage estimates of 65% and 31% for first and second dose (Table 2).

## Discussion

Across mainland Tanzania in 2007, 65% of women who had a pregnancy in the previous year reported receiving first dose of IPTp and 30% second dose. This national level analysis of individual, facility and policy influences on coverage has revealed that throughout the period 2005–2007 delivery of second dose was negatively influenced by facility level factors, and probably policy related factors in Tanzania, but not by the individual characteristics of women beyond living in an urban area.

A lack of association between uptake of two doses of IPTp and individual factors in Tanzania has been reported previously, right from the beginning of IPTp implementation. The Demographic and Health Survey conducted in 2004 – soon after nationwide IPTp implementation – found no individual level association with IPTp second dose, nor did a small-scale exploration during the same time-period [24,28]. Our finding of 65% first dose and 30% second dose coverage shows some improvement on the 2005 Demographic and Health Survey estimates, 53% and 22% respectively, but remains far from the target of 80%.

This analysis uses responses following the lead question "When you went to the clinic were you given the medicine to prevent malaria?" Restricting the data to only those women who could state the type of drug administered considerably restricts the number of valid responses as women frequently cannot state the drug used. However, the only drug administered as IPTp during the study period was SP. Observations from the facility survey suggest that stock outs of SP had a dramatic effect on coverage of the intervention. Previously the delivery of IPTp benefited from using the same drug as the first-line anti-malarial treatment drug, SP, and needed no separate delivery system. It would appear that since the change over to co-artemether lumifantrine in 2006, the mechanisms for maintaining stocks of SP for IPTp require urgent attention, particularly in non-urban settings. It is worth mentioning here that facility user data is prone to responder bias as participants tend to give more positive responses than in population level household surveys and therefore levels of coverage observed may not be comparable between the two sources. However, the finding that coverage was at least 100% higher for women who attended a clinic with SP in stock than women who attended a clinic with no SP is likely a true reflection of the problem.

Less than half of health education sessions observed delivered malaria prevention messages. Such observations are sometimes associated with a Hawthorne effect – that workers observed for a short period of time tend to improve performance – and so the reality may be lower. The finding is surprising in Tanzania where there is clear commitment to reducing the burden of malaria and a number of initiatives have been designed to raise the profile of malaria nationally. It is worthwhile to investigate why these training and support initiatives are not translating into measurable delivery of clear malaria prevention messages to clinic users.

The delivery of IPTp at outreach clinics was reported to have declined during the period of the surveys. Again, this finding is contrary to the strong national commitment to control malaria. It is possible that this has arisen because of drug shortages – with rationing of SP for clinic based services taking place. Certainly barriers to delivery of IPTp at outreach where the most remote and vulnerable women are targeted should be addressed.

The schedule for delivery of IPTp has been scrutinized over the last year in a number of settings [9,29-31]. The Tanzanian antenatal policy of giving IPTp first dose at the second antenatal visit and second dose at the third antenatal visit was developed in part for its simplicity, and to minimize the burden placed on health staff from the increasing number of interventions being channelled through antenatal clinics. However, for high coverage to be realized, this policy demands simultaneous behaviour change from the pregnant woman population – to first attend clinic earlier than the current median of 20 weeks gestation – and from the facility worker – to implement the schedule. A previous small scale study in Tanzania found that facility workers do aim to stay within the recommendation for IPTp delivery, and reported missed opportunities for protecting pregnant women with two doses [16]. The Tanzanian antenatal guideline is critically different from the current WHO recommendation to distribute SP to all pregnant women at the first visit *after quickening *[8]. This is also designed to be an easily operationalized policy which stays within safety limits of SP in pregnancy, and importantly is not dependent on individual timing preferences for first attendance to antenatal clinic. It may be that the Tanzanian IPTp antenatal schedule should be revised accordingly while there is no evidence of behaviour change for earlier first attendance to clinic.

Results from investigations on a smaller-scale in sub-Saharan Africa have indicated a combination of lack of awareness, health worker behaviour, stock-outs and policy as possible explanations for low recorded coverage of IPTp [16,18-20,32]. Our national level analysis indicates that, in Tanzania, successful delivery of at least two doses of IPTp relies on facility and policy level factors that could potentially be resolved. Following a trial of IPTp implementation in the community compared to routine clinics, Mbonye and others [33] found that the community approach resulted in higher – and earlier – two-dose coverage than the routine approach. However, they caution that it would be important to support access to routine care where all other access to antenatal services was focussed. In Zambia, where high coverage of IPTp second dose has been achieved and sustained within existing systems, an analysis of enabling factors highlights co-ordinated support to the routine clinic system and training to antenatal care workers as key enabling factors [22].

## Conclusion

Acting upon the observed rural stock outs and unintentionally restrictive antenatal guidelines in Tanzania is the most direct and sustainable option for optimising delivery of IPTp with SP. The challenge posed to IPTp with SP by increasing drug resistance of the *Plasmodium falciparum *parasite is beyond the operational control of the health system. Should a new drug for IPTp be recommended, the obstacles to high coverage will undoubtedly persist. It is essential that lessons are learned on how to maximize the existing regime in order to avoid any backward step on the road to 80% access.

## Competing interests

The authors declare that they have no competing interests.

## Authors' contributions

TM and KH participated in design and implementation of the study, data analysis and interpretation. RN and HM participated in design and implementation. CJ contributed to design of the study and interpretation of the findings. JB participated in design and data analysis. YS contributed to implementation of the study and interpretation of data. HS and JS supported activities at each stage. TM wrote the first draft of this manuscript. All authors contributed to, and approved, the manuscript for publication.
